# A loop-assisted inversion technique for easy removal of a gastric fundal tumor

**DOI:** 10.1055/a-2119-0999

**Published:** 2023-07-17

**Authors:** Zhang Tao, Long Chen, Jie Liu, Yi Ming Peng, Feng Ying Lin, Liang Sun, Jian Chen

**Affiliations:** Department of Gastroenterology, Nanchong Central Hospital, The Second Clinical Medical College, North Sichuan Medical College, Nanchong City, Sichuan, China

A 54-year-old woman presented with a stromal tumor (approximately 2 × 1 cm) in the gastric fundus ( Fig. 1 and Fig. 2 a ). After it had been marked and submucosal injection performed under endoscopic guidance, an electrosurgical knife was used to make a circular incision ( Video 1 ). This was challenging because of the difficult approach and the high risk of perforation, with an IT knife being used to make the incision ( Fig. 2 b ). A clip-anchored loop was fixed 1 cm from the incised wound ( Fig. 2 c ). A snare was then used to trap the incised mucosa and lift it, with the loop ring being slowly tightened ( Fig. 2 d ). After the snare was released, inversion of the tumor was observed ( Fig. 2 e and Fig. 3 ). Next, an electrosurgical knife was used to cut and expose the tumor margins, and a snare was then used to trap the tumor base and perform electrosurgical excision ( Fig. 2 f ). After the excision, the clean inverted wound was sutured using clips ( Fig. 2 g ). Finally, the loop was released. The resected specimen was an intact tumor measuring approximately 2 × 1 cm ( Fig. 4 ). At follow-up 1 month later, a flat wound with a residual loop was observed ( Fig. 5 ).

**Fig. 1:**
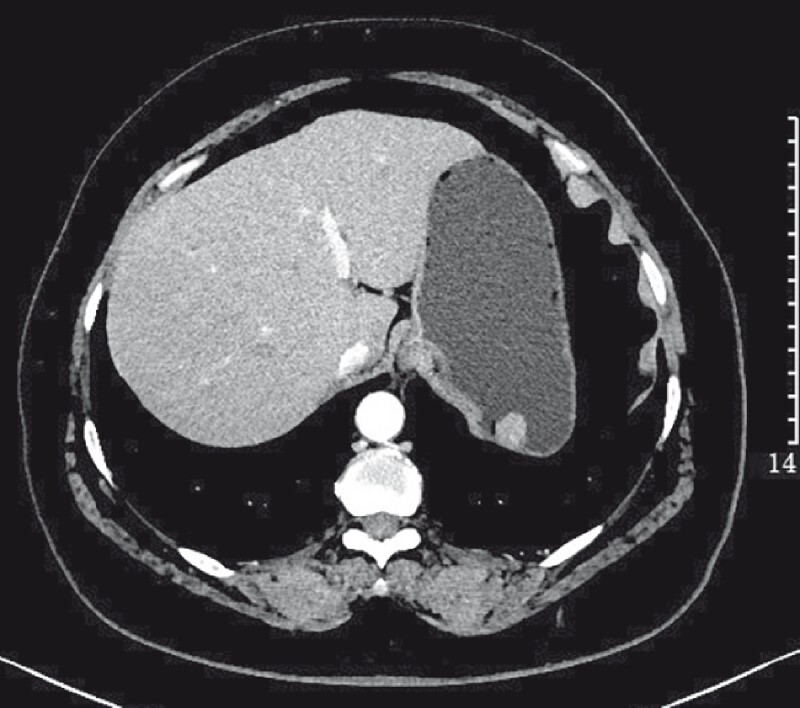
Computed tomography image showing a stromal tumor (about 2 × 1 cm) at the gastric fundus.

**Fig. 2:**
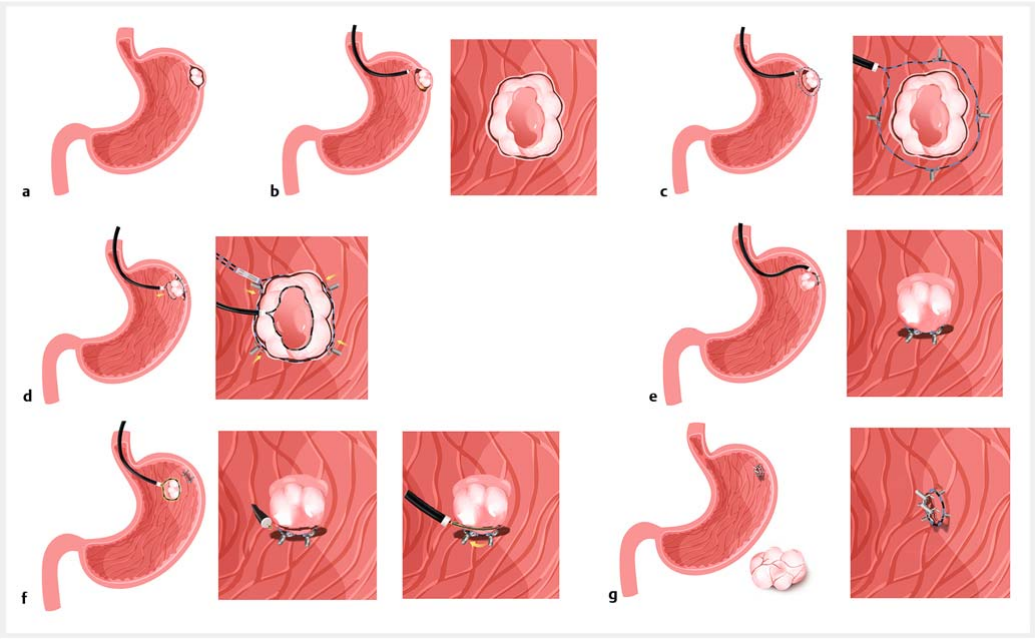
Schematic showing the stages involved in the procedure: **a** a tumor is present in the gastric fundus; **b** circumferential incision is performed after sufficient submucosal injection; **c** a loop is placed encircling the tumor and anchored by clips fixed 1 cm away from the incision; **d** a snare is used to entrap the incised mucosa and pull it towards the cardia, with the loop slowly tightened as the snare is pulled; **e** a protrusion that includes the tumor and normal tissue is seen after tightening of the loop and release of the snare; **f** an electrosurgical knife is used to cut and expose the tumor margins, then a snare placed around the margins of the tumor is used to trap and completely resect it by thermal snare cutting; **g** the internal wound surface is sutured, forming the double suture in combination with the loop ligation.

**Video 1** The loop-assisted inversion technique is performed to easily remove a gastric fundal tumor.


**Fig. 3:**
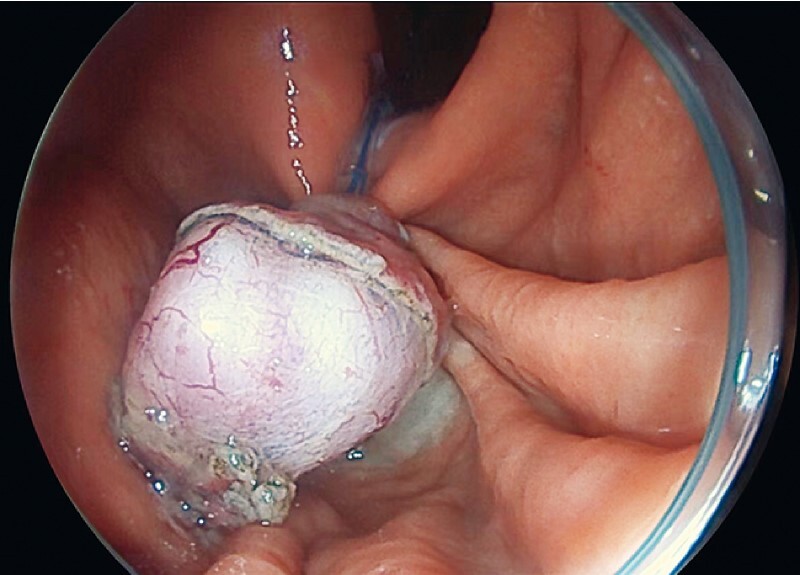
Endoscopic image showing the inverted tumor.

**Fig. 4:**
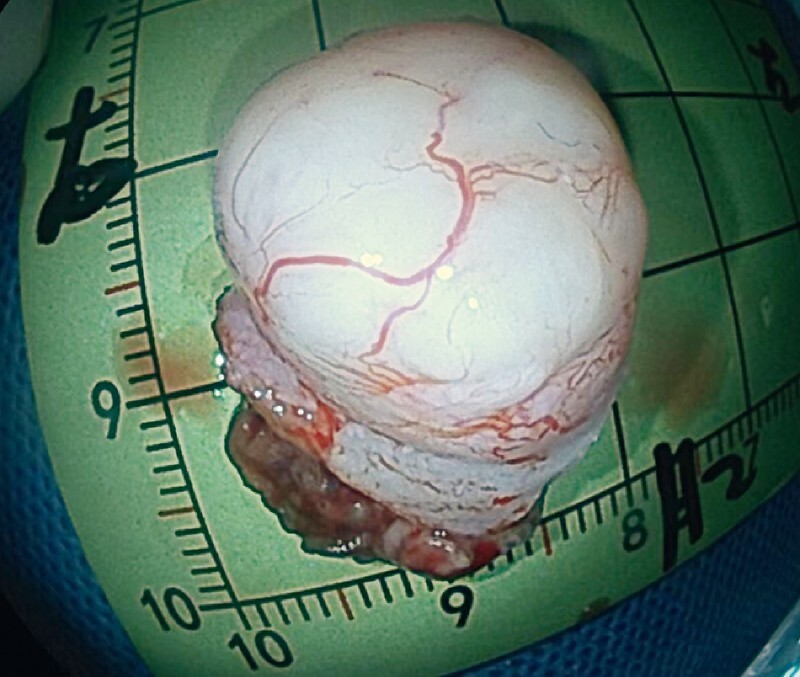
Photograph of the resected intact tumor.

**Fig. 5:**
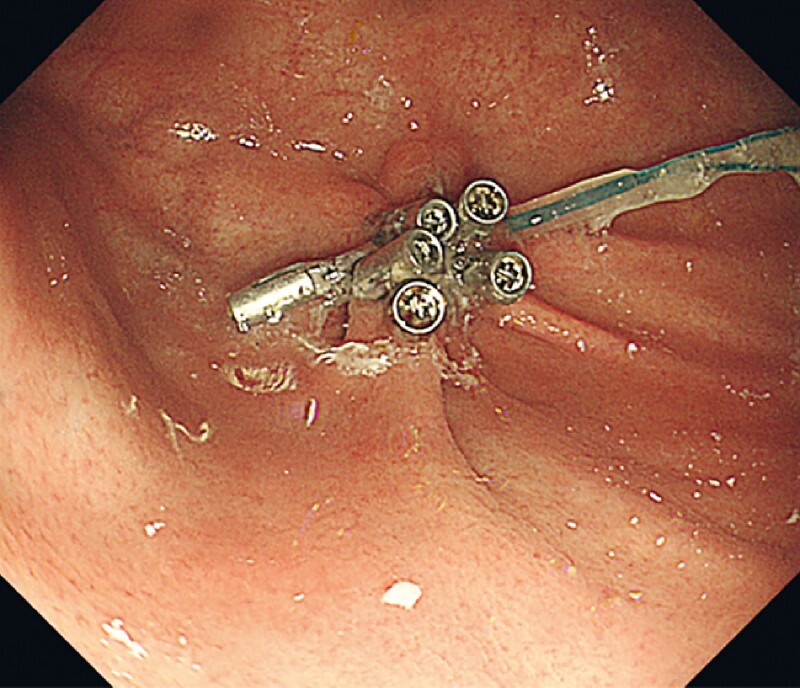
Endoscopic appearance at follow-up showing a flat wound closed by clips with the residual loop still in place after 1 month.

Endoscopic full-thickness resection (EFTR) is regularly used to treat gastric stromal tumors, is considered safe, and has a clinical outcome equivalent to surgery 1 . Gastric fundal tumors are associated with a high risk of perforation 2 . If perforation occurs, infection, intraperitoneal implantation metastasis, and postoperative bleeding of the serosal surface are potential concerns 3 . Several methods have been recommended for the management of unavoidable perforations 4 . We used a clip to fix the loop around the tumor and a snare to invert it. Double-suture techniques involving loops and clips are safe, easy, and quick.

Endoscopy_UCTN_Code_TTT_1AO_2AG

## Notes

Endoscopy E-Videos

https://eref.thieme.de/e-videos
*E-Videos* is an open access online section of the journal *Endoscopy* , reporting on interesting cases and new techniques in gastroenterological endoscopy. All papers include a high-quality video and are published with a Creative Commons CC-BY license. Endoscopy E-Videos qualify for HINARI discounts and waivers and eligibility is automatically checked during the submission process. We grant 100% waivers to articles whose corresponding authors are based in Group A countries and 50% waivers to those who are based in Group B countries as classified by Research4Life (see: https://www.research4life.org/access/eligibility/ ). This section has its own submission website at https://mc.manuscriptcentral.com/e-videos
