# Correction: A loop-assisted inversion technique for easy removal of a gastric stromal tumor in the fundus

**DOI:** 10.1055/a-2155-7468

**Published:** 2023-08-22

**Authors:** Zhang Tao, Long Chen, Jie Liu, Yi Ming Peng, Feng Ying Lin, Liang Sun, Jian Chen

**Affiliations:** Department of Gastroenterology, Nanchong Central Hospital, The Second Clinical Medical College, North Sichuan Medical College, Nanchong City, Sichuan, China

**Correction**

**Correction: A loop-assisted inversion technique for easy removal of a gastric stromal tumor in the fundus** Tao Z, Chen L, Liu J et al. A loop-assisted inversion technique for easy removal of a gastric stromal tumor in the fundus. Endoscopy 2023; 55: E902–E904, doi:10.1055/a-2119-0999 In the above-mentioned article, the title has been corrected. Correct is: A loop-assisted inversion technique for easy removal of a gastric stromal tumor in the fundus. This was corrected in the online version on August 22, 2023.

